# Empirical Investigation of the Structural Response of Super-Span Soil–Steel Arches During Backfilling

**DOI:** 10.3390/ma18153650

**Published:** 2025-08-03

**Authors:** Bartłomiej Kunecki

**Affiliations:** Faculty of Civil Engineering, Wroclaw University of Science and Technology, 27 Wybrzeże Stanisława Wyspiańskiego St., 50-370 Wrocław, Poland; bartlomiej.kunecki@pwr.edu.pl

**Keywords:** full-scale test, field tests, soil-steel structures, soil-steel composite bridge, strain gauges, deep corrugation, backfilling, design method, Swedish Design Method

## Abstract

This paper presents field investigations of a corrugated steel soil–steel arch structure with a span of 25.7 m and a rise of 9.0 m—currently the largest single-span structure of its kind in Europe. The structure, serving as a wildlife crossing along the DK16 expressway in northeastern Poland, was constructed using deep corrugated steel plates (500 mm× 237 mm) made from S315MC steel, without additional reinforcements such as stiffening ribs or geosynthetics. The study focused on monitoring the structural behavior during the critical backfilling phase. Displacements and strains were recorded using 34 electro-resistant strain gauges and a geodetic laser system at successive backfill levels, with particular attention to the loading stage at the crown. The measured results were compared with predictions based on the Swedish Design Method (SDM). The SDM equations did not accurately predict internal forces during backfilling. At the crown level, bending moments and axial forces were overestimated by approximately 69% and 152%, respectively. At the final backfill level, the SDM underestimated bending moments by 55% and overestimated axial forces by 90%. These findings highlight limitations of current design standards and emphasize the need for revised analytical models and long-term monitoring of large-span soil–steel structures.

## 1. Introduction

Structures made of corrugated steel plates have recently gained increasing popularity in transportation engineering as alternatives to conventional bridge systems. These buried structures, commonly referred to as soil–steel structures or soil–steel composite bridges, have been used in Europe for over 40 years. While smaller corrugated steel culverts are primarily employed in sewerage and drainage systems, larger-diameter culverts are increasingly used in the construction of bridges and viaducts [1,2,3,4,5,6].

Corrugated steel culverts have been implemented in various regions, including the United States, Australia, Canada, and Scandinavia, for applications such as underpasses, wildlife crossings [7], rockfall and avalanche protection [8], and road and railway tunnels [4,6,9]. The concept of long-span buried structures has evolved primarily through field experience, during which spans have gradually increased and the cover depth-to-span ratio has decreased. Initially, flexible soil–steel structures were built as riveted corrugated pipes with circular cross-sections and relatively short spans.

In 1993, a major advancement occurred with the introduction of deep corrugated steel plates, featuring a 381 mm pitch and a 140 mm depth profile. Designed to accommodate longer spans, higher soil covers, and low-rise shapes such as box culverts, these plates provide nine times the stiffness of traditional shallow corrugated plates. To overcome the limitations imposed by heavy live loads, large spans, and thick plate assemblies, a deeper corrugation profile (500 mm × 237 mm) was subsequently introduced. These enhanced plates offer high flexural capacity per unit area and improved performance in demanding soil–steel applications. Manufactured from steel with a minimum yield strength of 300 MPa and available in thicknesses ranging from 7 to 12.7 mm, these deeper corrugated plates meet the structural demands of modern infrastructure. These innovations have significantly expanded the capabilities and application range of corrugated steel structures [10].

Currently, the largest soil–steel corrugated structures exceed 25 m in span. Notable examples include a soil–steel arch bridge constructed along the DK16 expressway near the city of Ostróda in northeastern Poland (discussed later in this paper) and the Shamal Bridge in Dubai. The Shamal Bridge, consists of three buried soil–steel arches with spans of 32.40 m, 32.40 m, and 23.76 m. Utilizing the 500 mm × 237 mm deep corrugation profile, the structures were built with 12 mm thick plates (10 mm for the smaller structure). A 2.94 m soil cover above the crown was reinforced with dual layers of high-strength polyester geotextile. To limit lateral deformation during backfilling, the side zones were stabilized using 10 mm longitudinal and 8 mm transverse steel reinforcement bars embedded in the surrounding soil [11,12].

This paper presents the results of field tests conducted on a low-profile corrugated steel arch structure with a span exceeding 25 m, constructed without any reinforcement—representing the largest single-span arch of its kind built in Europe. The structure was previously described in [13], where non-destructive testing was conducted during the acceptance live load test using heavy vehicles, along with analyses and monitoring results collected one year after commissioning.

The primary objective of the present study is to document and analyze the internal forces and deformations recorded during the backfilling process of the aforementioned structure. The experimental data are compared with predictions obtained using the Swedish Design Method to evaluate its accuracy and applicability in the design of large-span soil–steel structures [13,14].

## 2. Description of the Tested Structure

A large-span soil–steel arch was constructed along the DK16 expressway near the city of Ostróda, in northeastern Poland. The structure consists of a low-profile corrugated steel arch with a span of 25.7 m and a rise of 9.0 m, measured at the structural axes. The arch was installed on two reinforced concrete foundations supported by Franki piles. The total length of the structure is 95.7 m, and it functions as a wildlife crossing beneath the expressway [13].

**Figure 1:**
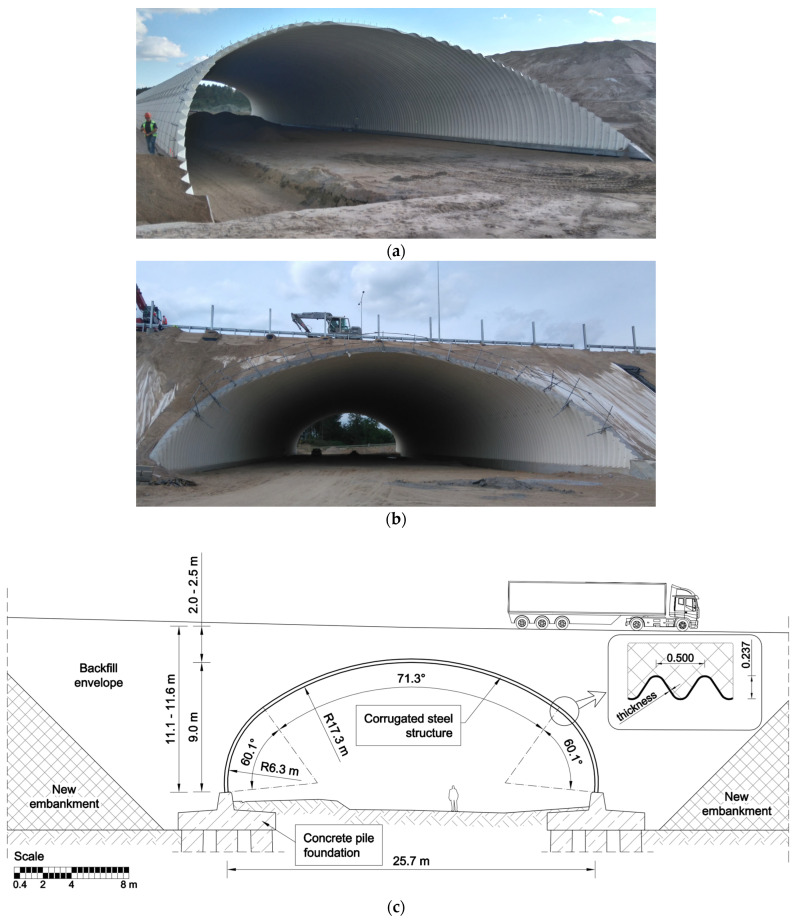
(**a**) View of the structure before backfilling, (**b**) the structure fully backfilled, and (**c**) geometry of the structure with the cross-section of the corrugated profile.

A schematic cross-section of the arch is presented in Figure 1c, while Figure 1a and Figure 1b show the arch before and after backfilling, respectively.

Steel grade S315MC, characterized by a minimum yield strength of 315 MPa for thicknesses up to 16 mm, was used in the production of corrugated plates. The steel material satisfied the requirements of European Standard EN 10149-2 [15]. The corrugation was 500 mm× 237 mm, currently the world’s deepest corrugation. Dimensions of a single corrugation are shown in Figure 1c and are defined in [16]. The steel plates were joined by ϕ 22 mm bolts. The cross-sectional properties of the corrugated profile are shown in Table 1.

## 3. Field Test

### 3.1. Backfilling Process

**Figure 2:**
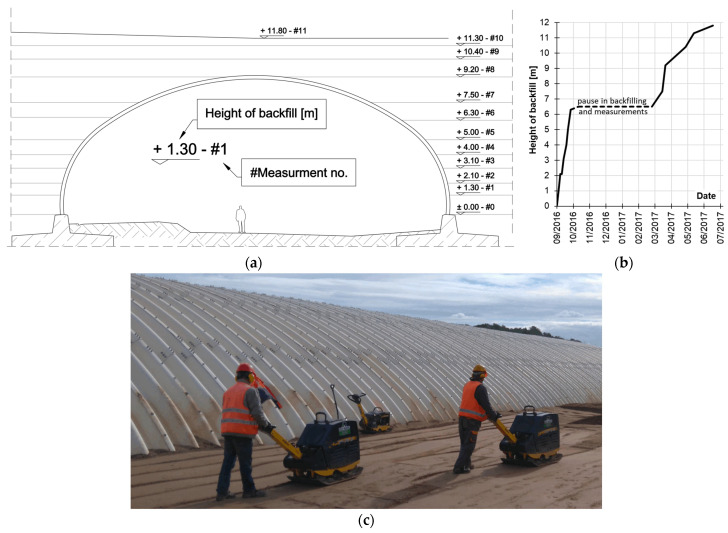
(**a**) Stages of backfill, where measurements were taken [m]; (**b**) the height of backfill in time with pause in backfilling and measurements; and (**c**) compaction process.

The structure was backfilled with soil to a height ranging from 2.055 m to 2.505 m above the crown. The embankment construction began on 10 September 2016. The first phase continued until 19 October 2016, reaching a backfill height of 6.3 m. The second phase resumed after a winter break on 3 March 2017. The embankment over the crown was completed on 26 May 2017, and the bituminous pavement was laid between 1 and 6 June 2017 [14]. The timeline and corresponding backfill elevations associated with the measurement campaigns are summarized in Figure 2a,b.

Backfill was placed and compacted symmetrically on both sides of the arch in loose lifts with a maximum thickness of 300 mm. The maximum differential height in backfill during construction did not exceed 300 mm. The maximum dry unit weight of the backfill material was 2.16 g/cm^3^, achieved at an optimum moisture content of 7.8% [14].

The compaction process along the structure’s side is shown in Figure 2c.

### 3.2. Instrumentation and Measurement

**Figure 3:**
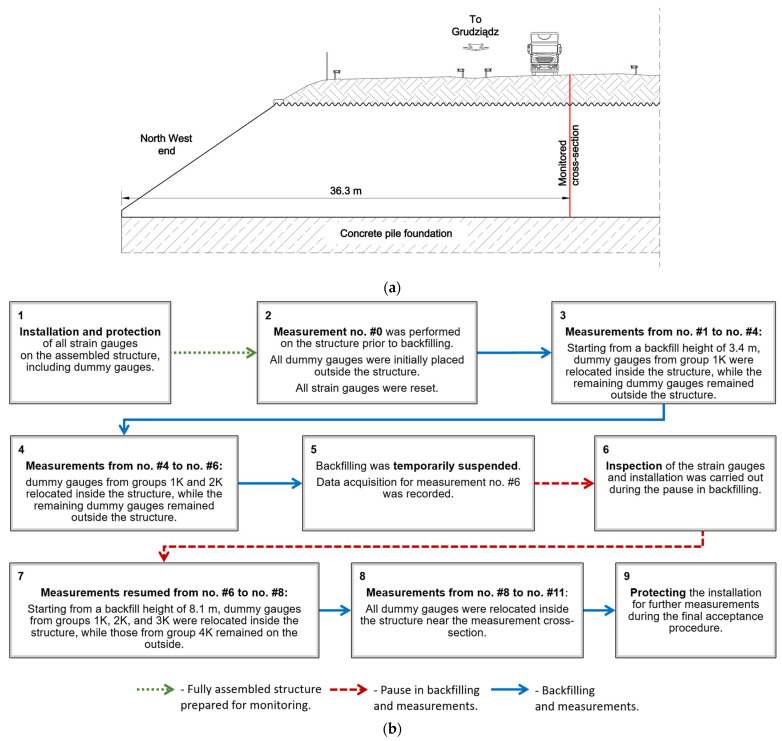
(**a**) Location of monitored cross-section and (**b**) block diagram of the strain measurement procedure.

The primary objective of the test was to monitor the structural behavior of a single corrugated steel arch during embankment construction. Measurements were performed after the backfill was placed on both sides of the arch at the levels indicated in Figure 2a. Deflections were recorded at nine locations, and strains were measured at 34 points on the steel plates throughout the backfilling stages. The data presented in this paper were collected on a ring located 36.3 m from the north-western end of the structure, as shown in Figure 3.

### 3.3. Strain Measurements

**Figure 4:**
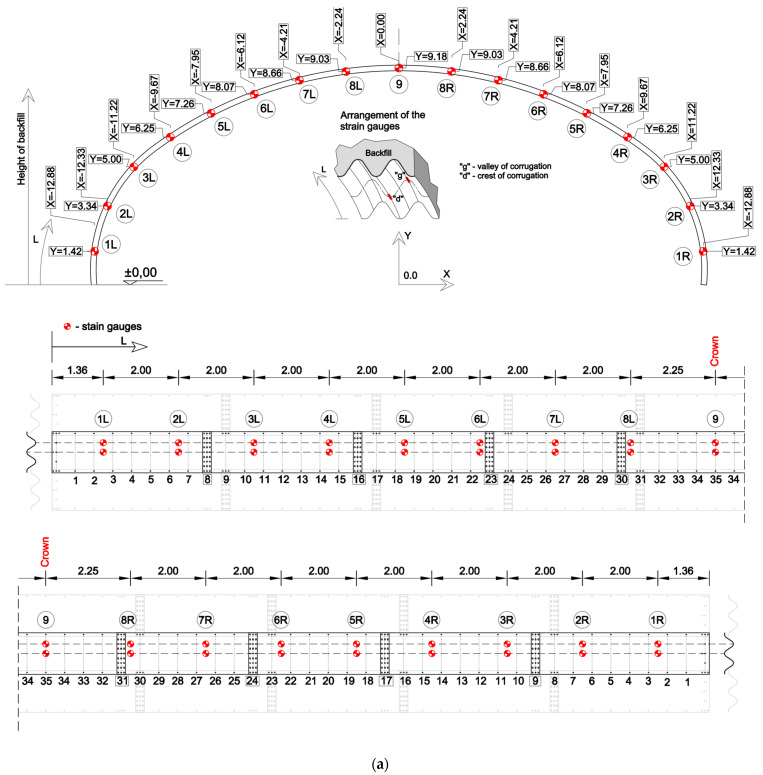

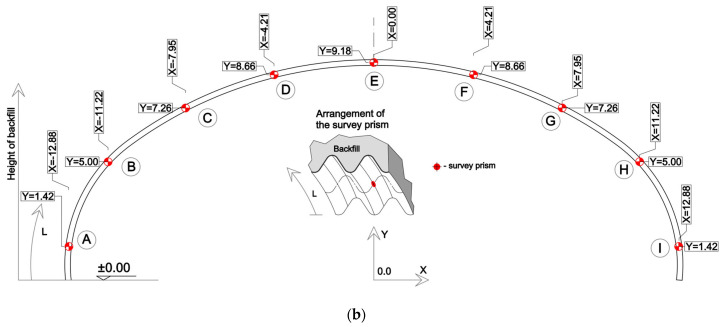
(**a**) Location of the strain gauges, and (**b**) location of the displacement gauges (targets).

Strain gauges were installed at 17 locations along the interior surface of the arch, directly beneath a traffic lane. At each location, two strain gauges were mounted—one at the crest and one at the valley of the corrugation—resulting in a total of 34 strain gauges and four dummy gauges. The layout of the strain gauge locations is presented in Figure 4a, which also shows their positions on the unfolded steel plate projection and their relative placement with respect to the longitudinal bolted connections.

**Figure 5:**
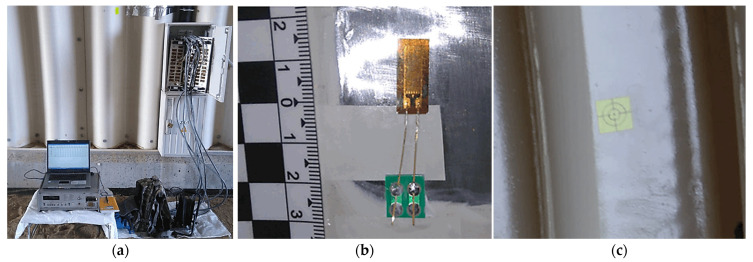
Measurement equipment: (**a**) the UPM 100 amplifier from Hottinger Baldwin Measurement (HBM) during strain measurement, (**b**) a typical strain gauge before protection, and (**c**) displacement gauges (target) installed on the corrugation.

Electro-resistant strain gauges with a 10 mm gauge length, 120 Ω resistance, and a gauge factor (k) of 2.02 were used. A typical strain gauge prior to waterproofing is shown in Figure 5b. Measurements were recorded using a UPM 100 data acquisition system from Hottinger Baldwin Messtechnik (Darmstadt, Germany)., connected to a computer equipped with the “Beam 1.0” software. The measurement setup at the construction site is presented in Figure 5a. All strain gauges were weatherproofed, and signal cables were routed through protective conduits securely fixed to the corrugated plates.

The 100-channel strain gauge amplifier used for the measurements allowed for the use of four additional dummy gauges, which were placed on unloaded steel elements and assigned to four appropriately distributed strain gauge groups (1K, 2K, 3K, 4K). This configuration minimizes the risk of measurement errors due to uneven solar heating of the structure during backfilling. The dummy gauges were assigned to specific groups of strain gauges as follows:

- Group 1K: 1L, 1R, 2L, 2R
- Group 2K: 3L, 3R, 4L, 4R
- Group 3K: 5L, 5R, 6L, 6R
- Group 4K: 7L, 7R, 8L, 8R, 9

Thermal effects required correction, particularly on sunny days when the structure was partially backfilled and the exposed steel surface experienced non-uniform heating. Prior to the start of the backfilling process, all dummy gauges were positioned outside the structure. As the backfill progressed and reached the level of specific measurement gauges, the corresponding dummy gauges were relocated inside the structure, near the measurement strip. Additionally, all measurements were conducted in the evenings after the completion of construction activities to minimize the influence of temperature variations caused by solar radiation and vibrations generated by construction equipment. To enhance the clarity of the strain measurement procedure, Figure 3b presents a block diagram that outlines the main stages of the measurements, including notes regarding the use and repositioning of dummy gauges.

### 3.4. Deflection Measurements

Three-dimensional deflections of the steel shell were measured during backfilling using a geodetic laser device (Leica Geosystems, Heerbrugg, Switzerland). A total of 45 measurement points were distributed across nine locations in five cross-sections of the arch. The deflection data presented in this paper were collected at the same ring (located 36.3 m from the north-western end of the structure) as the strain measurements.

Deflections at each point were determined by measuring changes in distance between fixed reference points on the ground and optical prisms mounted on the steel plates. Longitudinal deflections along the structure were found to be negligible and are not discussed further in this paper. The locations of measurement points along the corrugated plate and around the arch perimeter are shown in Figure 4b, and an example of a displacement target installed on the corrugation is shown in Figure 5c.

## 4. Test Results

### 4.1. Internal Forces Calculations

The strains measured at the valley of the corrugation ($\varepsilon_{g}$) and at the crest of the corrugation ($\varepsilon_{d}$) were used to calculate the corresponding stresses, $\sigma_{g}$ and $\sigma_{d}$, respectively. Stresses in the steel structure were determined from strain values, assuming a linear-elastic behavior with a Young’s modulus of E = 200 GPa. The following equations were applied for the stress calculations:

(1) $$\sigma_{g}=E\cdot \varepsilon_{g}$$

(2) $$\sigma_{d}=E\cdot \varepsilon_{d}$$

where

E —Young’s modulus,

$\sigma_{d}$—stress at the crest of corrugation,

$\sigma_{g}$—stress at the valley of corrugation,

$\varepsilon_{d}$—strain at the crest of corrugation, and

$\varepsilon_{g}$—strain at the valley of corrugation.

Section normal forces (N), also called thrust per unit width of structure, were calculated for a specific backfill level using the equation below:

(3) $$N=\frac{\left(\sigma_{d}+\sigma_{g}\right)\cdot A}{2}$$

where

A—cross-sectional area,

$\sigma_{d}$—stress at the crest of corrugation, and

$\sigma_{g}$—stress at the valley of corrugation.

Section moments (M) are calculated according to the following equations:

(4) $$M=\frac{\left(\sigma_{d}-\sigma_{g}\right)\cdot W}{2}$$

where

W—section modulus,

$\sigma_{d}$—stress at the crest of corrugation, and

$\sigma_{g}$—stress at the valley of corrugation.9

The calculations were carried out in accordance with the following sign convention: tensile strains and stresses, tension at the bottom of the cross-section, and upward displacements were considered positive.

### 4.2. Internal Forces Distribution

**Figure 6:**
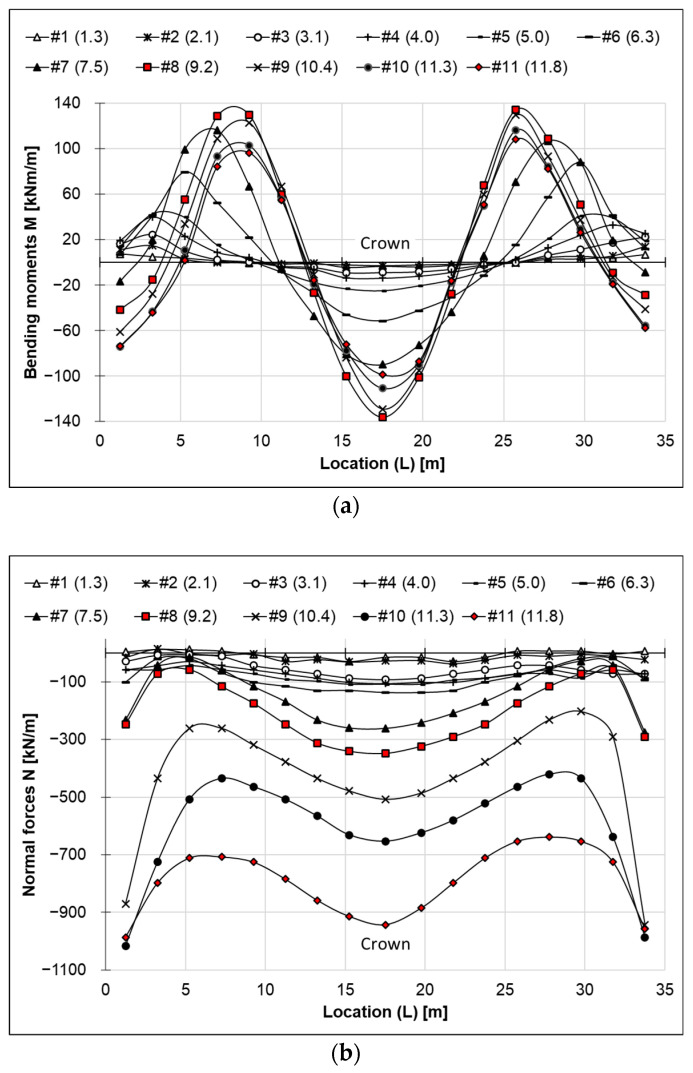
Distribution of: (**a**) bending moments vs. locations (L), and (**b**) normal forces vs. locations (L) (note: layer markings according to Figure 2a—the height of the backfill in [m] is given in brackets).

The distribution of internal forces is presented in two formats: bending moments versus the transverse arch length (L), and axial (normal) forces versus (L), where L denotes the length along the arch in the transverse direction. The distribution of bending moments is shown in Figure 6a, while the axial force distribution is shown in Figure 6b. In Figure 6, the measurement results obtained from the test are indicated by data points. For improved visualization, the points are connected by lines representing a prediction of the internal force distribution.

**Figure 7:**
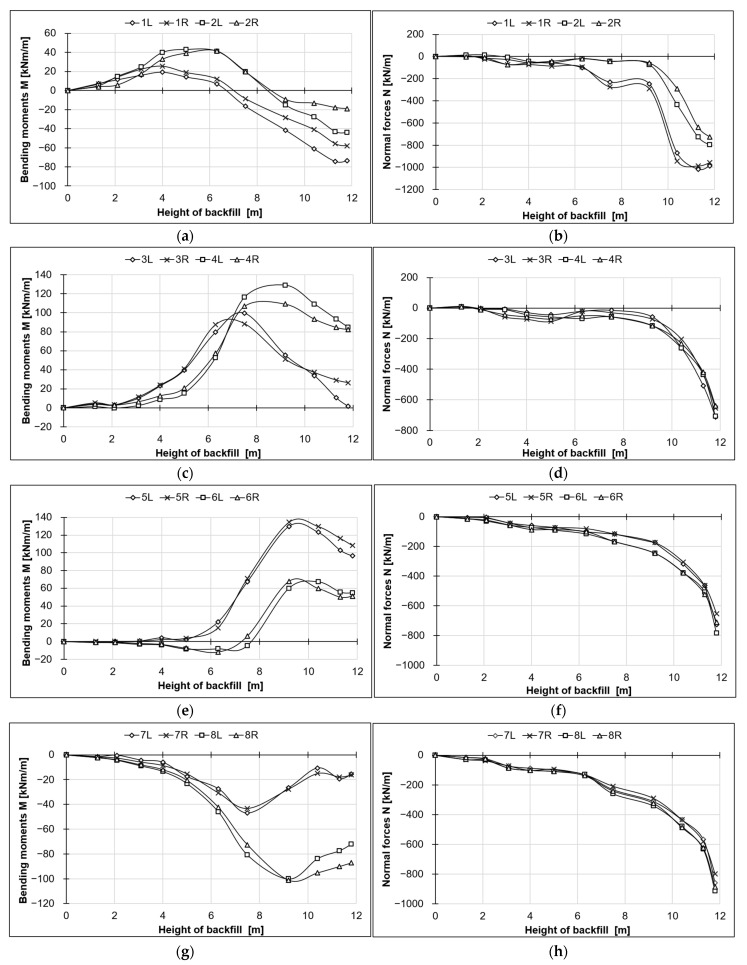

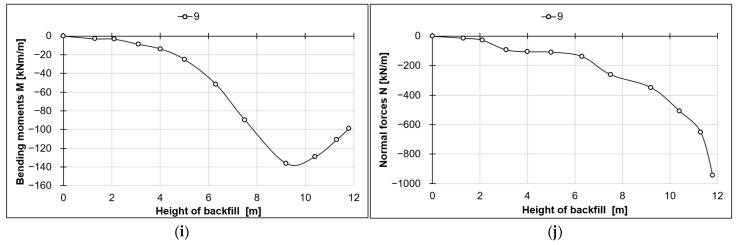
Bending moments and normal forces vs. height of backfill at: (**a**,**b**) points 1 and 2; (**c**,**d**) points 3 and 4; (**e**,**f**) points 5 and 6; (**g**,**h**) points 7 and 8; and (**i**,**j**) point 9.

Bending moments and axial forces (thrust) as functions of backfill height were analyzed symmetrically in pairs on the left and right sides of the structure and are presented in Figure 7. The analyzed point pairs include:

- Pair 1 and 2—Figure 7a,b;
- Pair 3 and 4—Figure 7c,d;
- Pair 5 and 6—Figure 7e,f;
- Pair 7 and 8—Figure 7g,h;
- Point 9 (crown) is presented separately—Figure 7i,j.

**Figure 8:**
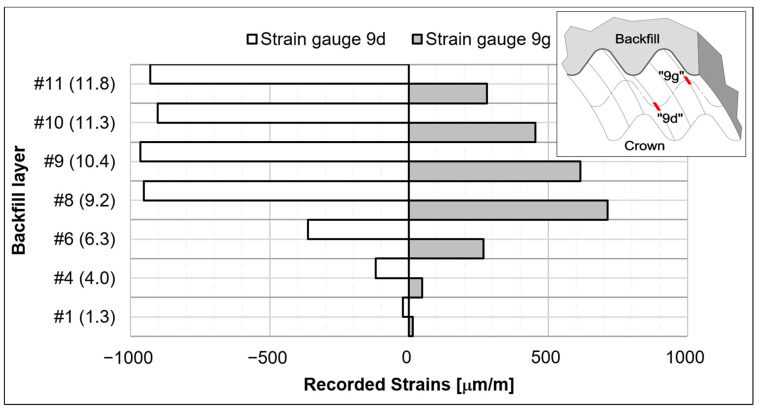
Recorded strains in the crown of steel arch (point 9) for selected level of backfill (note: the height of the backfill in meters is given in brackets).

The strains recorded at the crown (Point 9) are shown in Figure 8 and illustrate the structural response to increasing backfill height at selected levels.

### 4.3. Displacements

**Figure 9:**
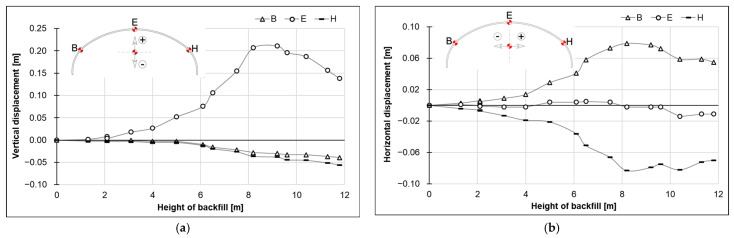
(**a**) Vertical displacements of the points: “B”, “E”, “H” vs. the height of backfill and (**b**) horizontal displacements of the points: “B”, “E”, “H” vs. the height of backfill.

Figure 9a presents vertical displacements as a function of backfill height at the crown and haunch locations (Points B, E, and H). Figure 9b presents the corresponding horizontal displacements at the same locations.

### 4.4. Summary of Test Results

Based on the measurements conducted during the backfilling process, the following conclusions and observations can be drawn:

- The minimum bending moment recorded during backfilling was −136 kNm/m and occurred at Point 9 (crown), when the backfill reached the crown elevation.
- The maximum bending moment recorded was 135 kNm/m and occurred at Point 5R (haunch), also when the backfill was near the crown elevation.
- The highest bending moments were observed when the backfill height reached the crown level. Comparable values were recorded at the crown and haunch zones.
- The minimum axial force (compressive) was −1016 kN/m and was registered at Point 1L after completion of backfilling.
- The maximum absolute strain recorded was 965 μm/m, occurring at the crown during the stage when the backfill was close to the crown elevation.
- The maximum upward displacement of the crown was 211 mm, which was also observed when the backfill was near the crown elevation.
- The maximum horizontal narrowing of the structure was 162 mm, recorded between Points H and B when the backfill height reached 8.6 m above the foundation level.
- A slight asymmetry in the internal forces was observed, corresponding to minor asymmetries in the recorded displacements.
- A reduction in bending moments and a simultaneous increase in axial forces were observed when the backfill height surpassed the crown elevation.
- It was observed that, with increasing backfill height, bending moments and axial forces exhibited divergent trends at measurement points symmetrically distributed along the arch cross-section.

For clarity, the extreme values of internal forces, stresses, and deformations are summarized in the tables below. Table 2 presents the maximum and minimum internal forces recorded during the test, including their values, locations, and the corresponding backfill heights.

Table 3 summarizes the extreme stress values recorded, along with their positions and related backfill elevations.

Finally, Table 4 presents the maximum displacements and deformations observed, including their locations and the backfill height at which they occurred.

## 5. Comparison with Design Method

### 5.1. Internal Forces During Backfilling

The Swedish Design Method (SDM) is a widely used soil–structure interaction design approach in Europe. Originally developed based on Swedish standards, the fifth edition of the SDM, updated in 2013, has been adapted to comply with Eurocode principles. According to the SDM manual, “the forces and moments described in this manual are calculated using the principles given in the so-called SCI method, Duncan (1978, 1979), with certain modifications regarding load distribution, soil modulus, depth of cover, and flatter culvert profiles” [17,18,19].

The SDM was calibrated for corrugation profiles with a pitch and depth of 381 mm × 140 mm, based on several full-scale tests. The maximum span of structures tested during the development of the method was approximately 14 m for the 381 mm × 140 mm profile.

It is important to note that the SDM has not been specifically calibrated for deeper corrugation profiles, such as 500 mm × 237 mm, nor for structures with spans exceeding the originally tested limits.

The SDM equations were used to calculate the predicted maximum strains according to the Swedish Design Method for two critical stages: when the backfill reached the crown level and at the final cover height [19].

Table 5 presents a comparison of internal forces obtained from field tests and those calculated using the equations from SDM. The comparison includes results for three backfill levels: 6.3 m, the crown level of the arch, and the final backfill level. Since the SDM does not provide formulas for predicting internal forces at backfill heights below the crown, only the experimental results are shown in Table 5 for the 6.3 m level, in order to illustrate the evolution of internal forces with increasing backfill height. The last row of the table presents the ratio between the values calculated using SDM and those measured during the test.

The results indicate that the equations used in the SDM do not accurately predict internal forces during the backfilling process, particularly when the backfill reaches the crown level of the arch. At the crown level, the calculated values overestimate the measured bending moments and axial forces by approximately 69% and 152%, respectively. At the final backfill level, the SDM underestimates the bending moment by about 55% and overestimates the axial force by approximately 90%.

Therefore, the fifth edition of the SDM is not recommended for the design of structures with 500 mm × 237 mm corrugation profiles and span exceeding 25 m. The field tests further confirm that the actual internal forces experienced by the structure during construction were notably lower than those predicted by the SDM. These findings underscore the limitations of the current SDM equations for large-span structures with deep corrugations.

Further research is recommended to refine existing design procedures and to complement them with advanced analytical or numerical methods as well as introduction of information on span and corrugation type limits when using SDM.

### 5.2. Deformations During Backfilling

Structural failure of soil–steel systems due to wall buckling can occur at deformation levels as low as 5% of the cross-sectional height [6]. Based on this observation, for safety assurance of corrugated steel soil–steel structures, it is generally accepted that the maximum permissible vertical or horizontal deformation should not exceed 2% of the structure’s height or span [1].

For the structure under investigation, 2% of the span corresponds to 514 mm, while 2% of the height equals 180 mm. Assuming a deformation limit of 180 mm, the measured maximum vertical deformation of the steel shell reached 211 mm (see Table 4 and Figure 9a), exceeding the threshold by 31 mm. In contrast, the horizontal deformation (narrowing) was 162 mm (see Table 4 and Figure 9b), remaining below the allowable limit. Despite exceeding the recommended vertical deformation limit, no damage to the steel shell was observed in the monitored cross-section, and the recorded stress levels did not exceed permissible values.

This comparison provides further evidence supporting the need to revise current design methodologies and recommendations for large-span soil–steel structures with deep corrugation profiles.

## 6. Summary and Conclusions

This paper presented the results of field measurements conducted on a large-span corrugated steel soil–steel arch structure during embankment construction. The tested arch, featuring a span of 25.7 m and a rise of 9.0 m, is the largest single-span arch of its kind built in Europe. Key findings and conclusions are summarized as follows:

- Maximum internal forces and displacements were observed during the backfilling phase, particularly when the fill reached and surpassed the crown level.
- Measured values of bending moments and axial forces were significantly different from those predicted by the SDM, with overestimations of 69% and 152%, respectively, at the crown level, and further inaccuracies at the final cover height.
- The results confirm that the fifth edition of the SDM, originally developed for shallower corrugations and shorter spans, is not suitable for structures with 500 mm × 237 mm corrugation profiles and spans exceeding 25 m.
- Maximum displacements and internal forces occurred during backfilling, underscoring the importance of monitoring throughout the construction process.
- A slight asymmetry in displacements and internal forces was observed, likely resulting from minor differences in construction conditions.
- The results indicate that even with a slight exceedance of the recommended deformation limit, the structural performance remained within safe bounds, highlighting the necessity to update design guidelines for large-span, deeply corrugated soil–steel structures.
- It was observed that with increasing backfill height, bending moments and axial forces exhibited divergent trends at various measurement points located symmetrically along the arch cross-section, as illustrated in the graphs in Figure 7. This behavior of internal forces can be attributed to the varying distances between individual pairs of strain gauges and the longitudinal bolted connections of the steel plates. These connections feature enlarged bolt holes to facilitate bolt installation, which allows for slight relative movement between the joined plates. Such localized displacements may influence the distribution of internal forces in the vicinity of the joints. A second potential cause of the divergent trends in internal forces with increasing backfill height is the possibility of asymmetric backfilling and compaction, or the use of a single work crew compacting the soil alternately on both sides of the arch.

Several field and laboratory tests have been carried out worldwide on soil–steel arch structures during the soil placement, including those described in references [20,21,22,23,24,25,26,27,28], some of which involved structures with deep corrugation profiles [11,12,13,14,29,30]. To the best of the author’s knowledge, structures with spans exceeding 25 m have been tested during backfilling in only two locations to date: the Shamal Bridge in Dubai and the arch structure near Ostróda in Poland, which is analyzed in detail in this paper. Therefore, the results presented in this study can support the verification of existing design methodologies and the calibration of numerical models for structures incorporating deep corrugation profiles, as well as guide the planning of future field tests for similar large-span structures.

Maximum displacements and internal forces in this type of structure typically occur during the backfilling process, which highlights the importance of structural monitoring during this construction phase. Similarly, long-term monitoring after construction is essential—especially for structures embedded in newly constructed embankments, where ongoing settlement may impose additional loads on the steel structure. Data presented in [13] indicate that permanent deformations in the analyzed structure were monitored for approximately one year following construction. The observed displacements were asymmetric and did not exceed 9 mm. Continued monitoring of this structure is strongly recommended.

The results emphasize the need for further development of analytical design methods tailored to current manufacturing capabilities and structural configurations. In particular, special attention should be given to the modeling of the backfilling phase. Long-term monitoring of similar large-span buried structures—especially those constructed in newly built embankments—is also strongly recommended, as post-construction settlements can introduce additional structural loads.

Recommendations for future research and analysis include:

- Updating analytical design methods and guidelines to reflect current manufacturing capabilities, with particular emphasis on modeling the backfilling phase.
- Conducting long-term deformation monitoring of buried soil–steel structures, especially those with spans exceeding 20 m and located within newly constructed embankments.

The presented experimental data can be used to calibrate and validate advanced numerical models and guide future development of design methods for large-span soil–steel composite bridges.

In summary, this paper presents experimental measurements of internal forces and displacements in the largest soil–steel structure in Europe (and, at the time of construction, the largest of its kind worldwide), erected without any additional reinforcements such as stiffening ribs, steel geogrids, or geotextiles. The measurements were carried out during the most critical phase of the structure’s lifecycle—backfilling. Based on the obtained results and comparative analyses, the study indicates that current analytical design methods and construction guidelines are outdated, particularly due to the lack of limitations regarding span length and corrugation size. The results underscore the necessity for continued monitoring of this type of structure throughout its operational lifespan.

## Figures and Tables

**Table 1:** Cross-section properties for corrugated profile.

Profile Dimension (mm × mm)	Cross-Section Area (mm^2^/mm)	Moment of Inertia (mm^4^/mm)	Section Modulus (mm^3^/mm)	Plate Thickness (mm)
500 × 237	14.51	96,733	817.69	9.65

**Table 2:** Summary of extreme internal forces.

	Maximum Banding Moment M_max_ (kNm/m)	Minimal Banding Moment M_min_ (kNm/m)	Maximum Normal Forces N_max_ (kN/m)	Minimal Normal Forces N_min_ (kN/m)
Registered value	135	−136	14	−1016
Point name (location)	5R (haunch)	9 (crown)	2L	1L
Height of backfill (m)	9.2	9.2	2.1	11.3

**Table 3:** Summary of extreme stresses.

	Maximum Stress (MPa)	Minimal Stress (MPa)
Registered value	153	−193
Point name (location)	5Rd (haunch)	9d (crown)
Height of backfill (m)	9.2	10.4

**Table 4:** Summary of extreme deformation.

	Maximum Uplift (mm)	Maximum Narrowing (mm)
Registered value	211	162
Point name (location)	9 (crown)	line B–H
Height of backfill (m)	9.2	8.6

**Table 5:** Internal forces at point 9 (crown).

Backfill Level	Backfill Level (6.3 m)		Crown Level (9.2 m)		Final Level (11.8 m)	
Internal Forces	Bending Moments (kNm/m)	Normal Forces (kN/m)	Bending Moments (kNm/m)	Normal Forces (kN/m)	Bending Moments (kNm/m)	Normal Forces (kN/m)
TEST	−52	−139	−137	−350	−99	−943
SDM	n/a	n/a	−230	−879	−44	−1793
Ratio: SDM/TEST	n/a	n/a	1.69	2.52	0.45	1.90

Note: TEST—Measured values; SDM—Swedish Design Method; n/a—not applicable.

## Data Availability

The original contributions presented in this study are included in the article. Further inquiries can be directed to the corresponding author.
